# Serum Lipidomics Meets Cardiac Magnetic Resonance Imaging: Profiling of Subjects at Risk of Dilated Cardiomyopathy

**DOI:** 10.1371/journal.pone.0015744

**Published:** 2011-01-20

**Authors:** Marko Sysi-Aho, Juha Koikkalainen, Tuulikki Seppänen-Laakso, Maija Kaartinen, Johanna Kuusisto, Keijo Peuhkurinen, Satu Kärkkäinen, Margareta Antila, Kirsi Lauerma, Eeva Reissell, Raija Jurkko, Jyrki Lötjönen, Tiina Heliö, Matej Orešič

**Affiliations:** 1 VTT Technical Research Centre of Finland, Espoo, Finland; 2 VTT Technical Research Centre of Finland, Tampere, Finland; 3 Helsinki University Central Hospital, Helsinki, Finland; 4 Kuopio University Hospital, Kuopio, Finland; 5 Helsinki Medical Imaging Center, Helsinki University Central Hospital, Helsinki, Finland; Istituto Dermopatico dell'Immacolata, Italy

## Abstract

Dilated cardiomyopathy (DCM), characterized by left ventricular dilatation and systolic dysfunction, constitutes a significant cause for heart failure, sudden cardiac death or need for heart transplantation. Lamin A/C gene (LMNA) on chromosome 1p12 is the most significant disease gene causing DCM and has been reported to cause 7–9% of DCM leading to cardiac transplantation. We have previously performed cardiac magnetic resonance imaging (MRI) to LMNA carriers to describe the early phenotype. Clinically, early recognition of subjects at risk of developing DCM would be important but is often difficult. Thus we have earlier used the MRI findings of these LMNA carriers for creating a model by which LMNA carriers could be identified from the controls at an asymptomatic stage. Some LMNA mutations may cause lipodystrophy. To characterize possible effects of LMNA mutations on lipid profile, we set out to apply global serum lipidomics using Ultra Performance Liquid Chromatography coupled to mass spectrometry in the same LMNA carriers, DCM patients without LMNA mutation and controls. All DCM patients, with or without LMNA mutation, differed from controls in regard to distinct serum lipidomic profile dominated by diminished odd-chain triglycerides and lipid ratios related to desaturation. Furthermore, we introduce a novel approach to identify associations between the molecular lipids from serum and the MR images from the LMNA carriers. The association analysis using dependency network and regression approaches also helped us to obtain novel insights into how the affected lipids might relate to cardiac shape and volume changes. Our study provides a framework for linking serum derived molecular markers not only with clinical endpoints, but also with the more subtle intermediate phenotypes, as derived from medical imaging, of potential pathophysiological relevance.

## Introduction

Dilated cardiomyopathy (DCM) is characterized by left ventricular dilatation and decreased contractility. DCM constitutes a significant cause for heart failure, sudden cardiac death or need for heart transplantation. At least 25% of DCM has been estimated to be familial [1]. The etiology, including genetic background is heterogeneous, with most disease-causing mutations accounting for a variable amount of DCM cases depending on the family and pedigree. So far the sole exception to this is the lamin A/C gene (LMNA) on chromosome 1p12 [2]. LMNA is the most significant disease gene causing DCM [3] and has been reported to cause up to 9% of DCM leading to heart transplantation [4]. LMNA codes both for lamin A and C proteins. A type lamins are expressed in most differentiated cells [5]. They are structural components of the nuclear lamina and involved in replication and transcription. At the cellular level, the mutations may affect the polymerization of the lamins, disrupt the structure of the nucleus and impair the function of chromatin. Clinically, mutations of the LMNA are associated with a large spectrum of human diseases called laminopathies, ranging from severe dilated cardiomyopathy to muscular dystrophy, progeria or lipodystrophy and altered plasma lipid levels [6], [7]. In women without known LMNA-mutations, low lamin A/C expression in adipose tissue has been reported to associate with increased fat cell lipolysis and altered lipid profile, including lower levels of high density lipoprotein cholesterol (HDL) [8]. The underlying disease mechanisms of LMNA mutations are a target of intensive research.

Clinically, identifying subjects in an early phase of developing DCM would be important but it is often difficult and for example subtle abnormalities in echocardiography may easily be overseen. We have previously shown that specific parameters derived from the heart cine magnetic resonance (MR) images help identify LMNA carriers from the controls at an early stage [9]. Here we apply global serum lipidomics in the same LMNA carriers and controls to examine possible effects of LMNA mutations to serum lipid profiles. Secondly, a novel visualization model was created to predict the possible effects of changes in these lipid parameters to left ventricular function as assessed by cardiac MRI.

## Results

### Study design

**Figure 1:**
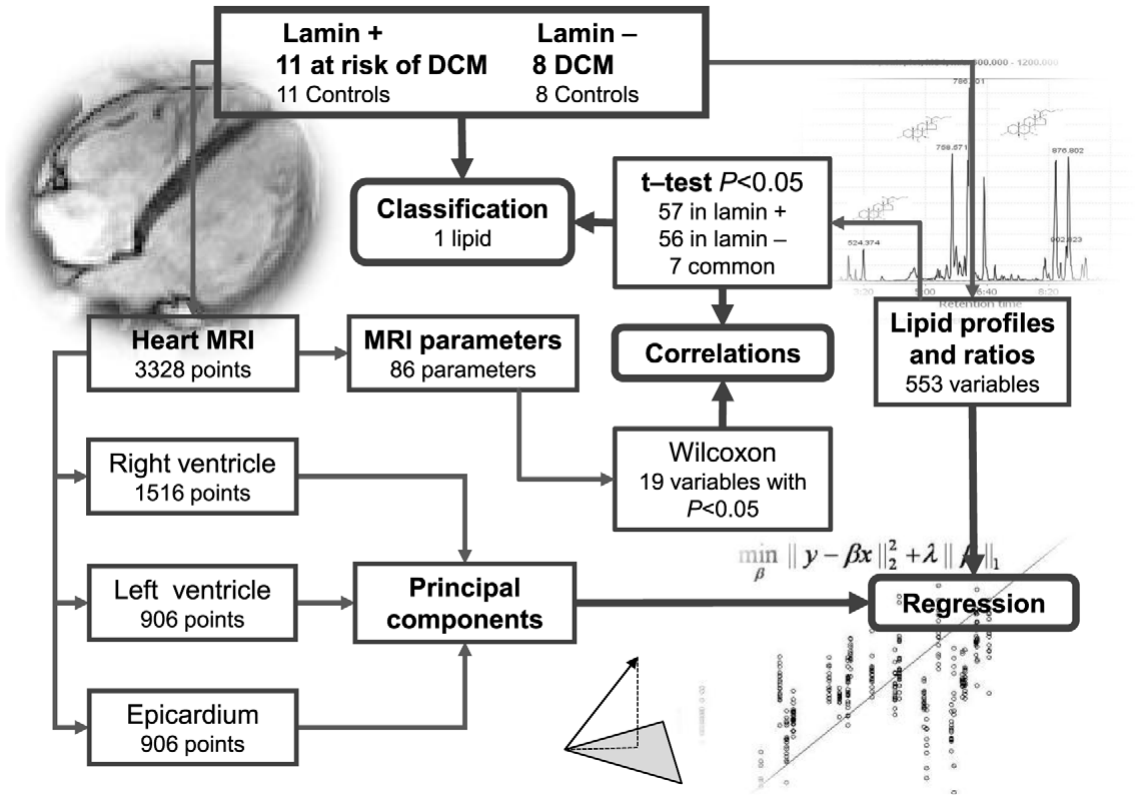
Study design, methods, and outcomes.

We first investigated the differences in serum lipidomes and cine MR image data between the LMNA carriers not diagnosed with DCM, and matched healthy controls (Figure 1). The “Lamin+” study included two groups: (Group 1) 11 LMNA mutation carriers at high risk of DCM, who were at the time of analysis not diagnosed with DCM, and (Group 2) 11 healthy controls that were age and gender matched to Group 1. Next, we investigated which of the molecular changes from the first study are generalized to DCM and are thus not specifically associated with the LMNA mutation. The second study (“Lamin−“ study), was performed independently, to provide control plasma samples from LMNA-mutation negative DCM patients for lipidomic analyses. It included also two groups: (Group 3) 8 diagnosed DCM patients without the LMNA mutation, and (Group 4) 8 healthy controls that were gender matched to Group 3. Clinical summary of the study participants is shown in Table 1.

**Table 1:** Summary of the study subjects included in the study. *P<0.1; **P<0.05.

	Lamin+ Study		Lamin− Study	
Group	1	2	3	4
Description	LMNA mutation carriers	Controls of Group 1	DCM-diagnosed subjects	Controls of Group 3
Men/women	4/7	4/7	3/5	3/5
Age (years)	33,7 (11,9)	34,3 (10,2)	56,4 (11,3)**	35,8 (10,3)**
BMI (kg/m2)	23,9 (4,2)	24,5 (2,6)	28,3 (2,8)*	24,2 (4,9)*
**Cardiac symptoms**				
chest pain	0	0	0	0
dyspnoea	1	0	5	0
palpitation	2	0	1	0
Cardiac diagnosis	1	0	8	0
none	0	11	0	8
healthy mutation carrier	10	0	0	0
DCM	0	0	8	0
Atrial fibrillation	1	0	0	0
Hypercholesterolemia	0	1	1	0
**Cardiac medication**	1	1	8	0
ACEinhibitor/ATRblocker	0	0	8	0
Betablocker	1	0	8	0
Digoxin	0	0	1	0
Diuretic	0	0	6	0
Statin	0	1	1	0
Warfarin	1	0	1	0
**LMNA mutations**	11	not determined	0	not determined
Ser143Pro	8			
Ala132Pro	2			
T1085Xdel	1			

We applied Ultra Performance Liquid Chromatography coupled to mass spectrometry (UPLC/MS™) to study molecular lipids as previously described [10]. A total of 286 lipids were identified across all four groups and included in the analysis. In addition, we also calculated ratios of specific lipids reflecting enzyme activities of fatty acid desaturation and elongation, which contributed additional 267 variables. The lipid variables were then log-transformed to make their distribution more Gaussian. In total, the final lipidomics dataset comprised 553 variables measured across all 38 subjects.

The cine MR images were analyzed from 11 LMNA carriers and their 11 matched controls (Groups 1 and 2). Surface models of the epicardium (1516 points) and the endocardium of the left and right ventricles (906 points each) containing all together 3328 points were mapped on the heart images. The heart surface models were also used to derive 86 variables that describe anatomical and physiological features of the heart, as described earlier [9].

### LMNA subjects have a distinct serum lipidomic profile

A total of 57 and 56 lipid variables were found significantly different when comparing Groups 1&2 and Groups 3&4, respectively (Table S1). Comparison of profiles of LMNA carriers (Group 1) and their controls (Group 2) revealed differences dominated by several phosphatidylethanolamines, with the direction of change depending on the fatty acid composition, as well as by diminished odd-chain triglycerides and lipid ratios related to desaturation (*i.e.*, increase of double bonds). Since LMNA mutations are known to be associated with lipid metabolism [11], the detected differences may be specific to LMNA mutation but may not be directly relevant to the risk of DCM. In a follow-up study we therefore investigated subjects diagnosed with DCM, but without LMNA mutation (Group 3), and controls which were not matched for age (Group 4). Comparison of the results from the two studies revealed seven common molecular lipids which were differentially regulated in LMNA mutation carriers as well as in subjects diagnosed with DCM (Table 2).

**Table 2:** Summary of between-group lipid changes in the two studies (Lamin+ and Lamin−).

	Lamin	Group	*P*-value	Fold	25%	50%	75%
**PC(38∶5e)**	+	**1**	0,0426	0,7	0,50	0,60	0,84
	+	**2**			0,82	0,85	0,96
	−	**3**	0,0307	0,7	0,46	0,50	0,54
	−	**4**			0,65	0,72	1,03
**PS(38∶2)**	+	**1**	0,0080	0,6	2,07	2,48	2,78
	+	**2**			2,88	4,38	5,05
	−	**3**	0,0348	0,7	1,05	1,29	1,57
	−	**4**			1,50	1,86	2,33
**TG(49∶2)/TG(49∶1)**	+	**1**	0,0194	0,9	0,61	0,73	0,79
	+	**2**			0,83	0,86	0,92
	−	**3**	0,0011	0,6	0,51	0,56	0,65
	−	**4**			0,90	0,96	1,07
**TG(49∶3)**	+	**1**	0,0002	0,5	1,70	2,05	2,62
	+	**2**			3,49	4,08	4,34
	−	**3**	0,0212	0,6	1,56	2,13	2,57
	−	**4**			3,04	3,68	4,70
**TG(50∶10)**	+	**1**	0,0195	0,8	0,66	1,27	1,69
	+	**2**			1,53	1,69	2,11
	−	**3**	0,0104	0,6	1,77	2,17	2,77
	−	**4**			3,04	3,68	4,70
**TG(50∶2)/TG(50∶1)**	+	**1**	0,0151	0,5	0,02	0,03	0,03
	+	**2**			0,04	0,06	0,10
	−	**3**	0,0025	0,1	0,00	0,01	0,01
	−	**4**			0,03	0,05	0,06
**TG(54∶3)/TG(54∶2)**	+	**1**	0,0359	0,8	3,24	4,49	5,15
	+	**2**			4,33	5,87	6,52
	−	**3**	0,0004	0,4	1,74	2,05	2,61
	−	**4**			3,98	5,16	5,48

Each lipid was separately compared between Groups 1&2 and 3&4 using the two-sided *t*-test. Reported fold change is the median of Group 1 or Group 3 divided by the median of Group 2 or Group 4, respectively. Also shown are the 25%, 50% and 75% quantiles.

**Figure 2:**
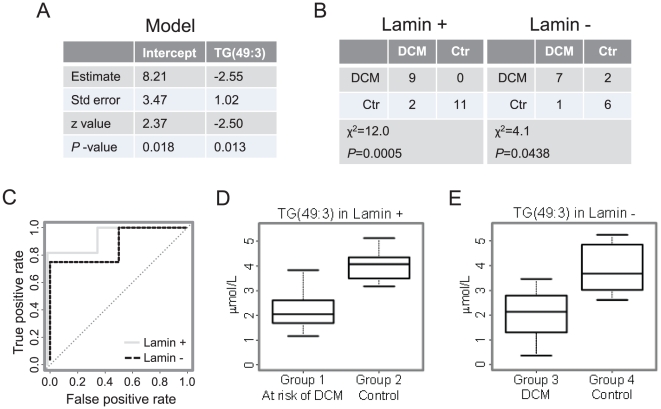
Logistic regression model using only one lipid variable, TG(49∶3), moderately discriminates subjects at risk of DCM from the controls. The model was trained using samples from Lamin+ study (LMNA mutation carriers and their controls) and applied to samples from Lamin− (DCM patients without LMNA mutation and their controls). (A) Parameter estimates, their standard errors and the z- and p-values of the estimates of the logistic regression model. Negative parameter estimate indicates that the risk of DCM increases with lowering concentrations of TG(49∶3). (B) Model performance and (C) and ROC curves in Lamin+ and Lamin− studies. Lower levels of TG(49∶3) do not depend on the lamin mutation (panel D) but are DCM specific (panel E).

In order to further investigate the disease specificity of lipidomic changes detected in LMNA mutation carriers, we used the lipids from Table 2 to fit a logistic regression model to samples from Groups 1&2 with the LMNA mutation (elevated DCM risk) as the response variable. The best subset of lipids was selected by the stepwise AIC algorithm [12]. The classification threshold of the regression model was tuned to minimize the number of misclassifications. The final model included only one variable, the odd-chain triglyceride TG(49∶3) (Figure 2A). The same model applied to samples from Groups 3&4 performed reasonably well (*P* = 0.044 by Chi-square test; Figure 2B–E), suggesting that the marker is not due to LMNA mutation but is DCM specific.

### Lipidomic profile from LMNA mutation carriers associates with specific cardiac shape and volume changes

**Figure 3:**
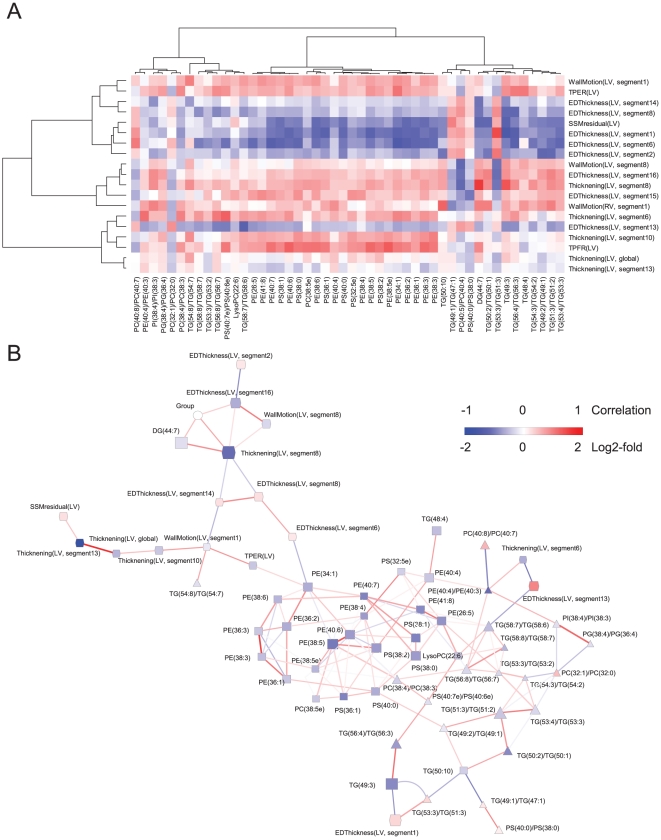
Associations between the lipid profiles and the MR image parameters. (A) Heatmap of Pearson's correlations between the lipid profiles and the MR image parameters. (B) Partial correlation graph. An edge denotes partial correlation between the nodes it connects. Width of an edge is proportional to the inverse of the non-rejection rate [13], which indicates the confidence with which the hypothesis of null partial correlation is not rejected, that is, the chance of the edge not existing in the graph is the smaller the smaller the non-rejection rate is. A threshold of 0.53 was used for the non-rejection rate: edges with higher values were omitted from the graph.

Next, we investigated in the group of LMNA mutation carriers if and how are the detected lipid changes reflected in cardiac shape and volume changes as measured by cine MR imaging. The MRI-derived parameters were compared between the Groups 1 and 2 and 19 variables out of 86 were found to be different (*P*<0.05 by Wilcoxon test). Linear correlation analysis between these image variables and the 57 significantly changing lipids (Table S1) revealed a large degree of positive or negative associations (Figure 3A), suggesting the serum lipidomic profile does reflect the changes in the heart shape and volume of the LMNA mutation carriers. To further study the specific dependencies of these variables, we applied the undirected Gaussian graphical Markov model which has been previously applied to study gene regulatory network from small sample sets [13] (Figure 3B). Such an approach aims at deriving the partial correlations between two variables conditioning on the remaining ones, and building a network where the variables are connected by an edge if and only if their partial correlation is significantly non-zero. Unlike the Pearson correlation coefficients, use of partial correlation adjusts for the confounding effects and thus removes spurious associations to a large extent. This is particularly favorable when dealing with high throughput data as it discovers only those direct interactions with high confidence. The variables formed two major clusters. One cluster is dominated by MRI-derived variables and the other by lipid variables. As an exception, the section of the lipid cluster which is associated with LMNA mutation as well as with DCM (as shown in Table 2) was associated with EDThickness (LV, segment1) (Figure 3B). This MR image parameter was increased in LMNA mutation carriers and negatively correlated with most of the molecular lipids found diminished in that group (Figure 3A).

We then investigated how the observed changes in lipids associate with the anatomy of the heart. We reconstructed a mean heart model and studied its sensitivity to specific lipid profiles. Heart surface models were derived from the heart MR images for the samples from Groups 1&2. The analysis was performed for each surface point by regressing the lipid profiles to the heart surface models' deviations from the mean heart model. To regularize the analysis, only the deviations on mean heart model's surface normal were studied. To illustrate the effect of a selected lipid on the heart surface model, the mean value of the lipid was computed for the Group 2, and the heart surface model corresponding to the mean lipid value was generated from the regression models of each surface point. Then, the lipid value was either increased or decreased (two times the standard deviation in Group 2) according to the change observed in Group 1, and the resulting heart model was generated. The change in the heart model was visually evaluated by computing the wall thickening and/or wall motion from end-diastolic shape to end-systolic shape and visualizing the results using color maps overlaid on the mean heart model.

**Figure 4:**
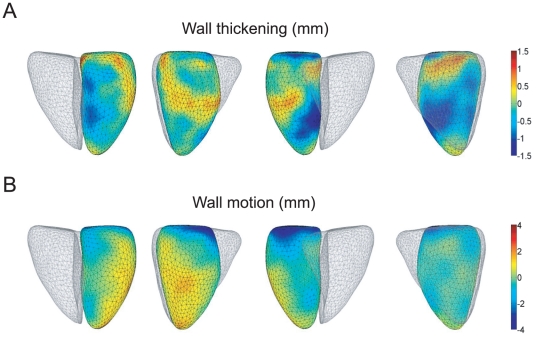
The effect of TG(49∶3) on the wall thickening and wall motion of left ventricle. The difference in wall thickening in mm (Panel A) and wall motion in mm (Panel B) between the mean lipid value of Group 2 and the decreased lipid value. The blue color indicates the regions where the wall thickening/motion decreased when the TG(49∶3) value was decreased. Therefore, the decreased function in these regions is associated with the LMNA-related TG(49∶3) concentration change. Respectively, red color indicates the regions that showed increased wall thickening/motion related to the TG(49∶3) concentration decrease in LMNA mutation.

Figure 4 shows the sensitivity of the heart model to change in TG(49∶3) concentration, *i.e.* a lipid found diminished in LMNA mutation carriers as well as in DCM (Table 2 and Figure 2). Figure 4A shows the change in wall thickening. Blue color indicates locations of reduced wall thickening as lipid concentration was decreased whereas red indicates increased wall thickening. Figure 4B shows the corresponding results for wall motion. Decrease of lipid concentration caused decrease in wall thickening and motion in septum. Also, reduced wall motion in base was observed.

## Discussion

In this preliminary study we revealed that LMNA carriers at risk of DCM are characterized by a distinct serum lipidomic profile as compared to apparently healthy controls. The association analysis using dependency network and regression approaches also helped us to obtain novel insights into how the affected lipids relate to cardiac shape and volume changes and confirm their disease specificity.

Diminishment of odd-chain triglyceride in LMNA carriers was associated with increased end-diastolic wall thickness in left ventricle (Figure 3B) but the meaning of this finding is unclear. Asynchrony might cause increased values when measurements are made gated by ECG. Projection of the lipid change onto the surface normal of the mean heart model was accompanied with the decrease in wall thickening and motion in septum. This is in line with earlier observations which show that DCM caused by LMNA mutations is characterized by diminishing left ventricular contractility while simultaneously the left ventricular end diastolic dimension may enlarge only modestly or does not fulfill formal criteria of DCM at all [14]. Among the affected lipids, the diminished odd-chain triglycerides are of particular interest as they were also diminished in DCM independent of LMNA mutation and thus could be considered as DCM risk marker candidates but this warrants further studies. Odd-chain triglycerides have been previously associated with mitochondrial fat oxidation disorders and their dietary supplementation has led to promising results in the treatment of chronic cardiomyopathy, rhabdomyolysis, and muscle weakness [15].

Odd-chain fatty acids are not endogenously synthesized in the body but are abundant in some diets such as milk. In fact C15∶0 and C17∶0 fatty acids have been considered as biomarkers of dietary milk fat intake [16]. Given the experimental setting of our study, the diet itself is an unlikely factor behind the observed diminishment of odd-chain triglycerides in the DCM groups. Instead, the altered absorption of lipids or formation of triglycerides from fatty acids in enterocytes are more plausible factors. The potential roles of intestinal lipid uptake and formation in the context of DCM are so far unexplored and there is currently no evidence that the common Finnish LMNA mutations associate with the intestinal lipid metabolism.

In order to characterize the molecular and physiological disturbances during the prodromal phases of the disease of relatively low incidence in general population, large prospective studies are needed with long term and frequent follow-up. This is in practice rarely possible. An alternative strategy applied also in this study is to identify subjects at high risk of disease, *e.g.* specific risk mutation carriers, and follow them up during the asymptomatic phase. In such case, additional studies may be needed to demonstrate the disease-specificity of early molecular or phenotypic changes. The potential shortcoming of such a strategy is that in many cases the study sample is small due to rarity of mutations in the general population, and dealing with relatively small sample sizes is an inherent statistical challenge that needs to be carefully addressed. The LMNA carriers in this study mainly carry the Ser143Pro mutation which is typical for the Finnish population. All the mutations which these patients carry are known to associate with cardiomyopathy later in middle age. The limitation of our study is the small sample size of LMNA mutation carriers. However, for modeling purposes, all available mutation carriers were recruited to obtain a relatively homogeneous group of subjects at risk of developing DCM.

In conclusion, using a novel integrative approach by combining serum lipidomics and MR imaging in LMNA mutation carriers at risk of DCM, we have shown that specific lipid abnormalities such as diminished odd-chain triglycerides precede DCM. Our findings should be considered preliminary and the possible role of odd-chain triglycerides in the development or prevention of DCM warrant further investigations. On a general level, our study also provides a framework for linking serum derived molecular markers not only with clinical endpoints, but also with the more subtle intermediate phenotypes, as derived from medical imaging, of potential pathophysiological relevance.

## Materials and Methods

### Study Participants

The *LMNA* mutations had initially been identified among DCM patients who participated in the molecular genetics study of DCM carried out in collaboration between the Helsinki University Hospital and the University of Kuopio [4], [14]. Group one comprised eleven mainly asymptomatic *LMNA* mutation carriers from these families recruited between years 2004–2006. The participants had not yet developed DCM by the time of this study but it is known that the penetrance of *LMNA* mutations is high [17]. None of these individuals had symptoms of skeletal muscle disease. Cardiac MRI was performed and venous blood samples obtained during years 2004–2006.

The second group consists of healthy controls matched to group 1: 11 individuals (four men and seven females) who did not have any cardiac disease, recruited during year 2007. Clinical examination, ECG and echocardiography (2-dimensional, M-mode and Doppler) were performed to each of the participants to exclude the possibility of cardiomyopathy or valvular disease. Venous blood samples were drawn for lipid analyses. DNA analyses were not performed.

Group three comprises eight unrelated DCM patients with no LMNA mutation (three men and five women), who had been diagnosed with DCM and followed up at the Department of Cardiology of the Helsinki University Hospital. The diagnosis was based on clinical examination, ECG and echocardiography. Clinical data was obtained from the hospital records. Inclusion criteria were LVEDD>27mm/m^2^ and EF<45% at time of diagnosis. All patients who had secondary causes of DCM were excluded. The subjects were asked to participate in the molecular genetics study of DCM during the years 2000–2008. In some patients the left ventricular function improved remarkably during medication. In the gene analyses carried out either by SSCP (single strand conformation polymorhism) -method or sequencing of the *LMNA* gene at the University of Kuopio, no mutations in the lamin A/C gene could be detected in these patients. Venous blood samples were drawn for lipid and metabolomic analyses and cardiac MRI studies were performed in year 2008.

Group four consists of healthy controls, matched for age and sex with group three: eight previously healthy volunteers (three men and five women), recruited in 2008. The possibility of cardiomyopathy or valvular disease was excluded by clinical examination, ECG and echocardiography (2-dimensional, M-mode and Doppler). Venous blood samples were drawn for lipid analyses. DNA analyses were not performed.

Informed consent was obtained from the study participants, and the Ethics review Committee of the Department of Medicine, University of Helsinki, approved this study.

### MR Imaging

The imaging was done as described previously [9]. In short, patients were evaluated by personal and family history, physical examination, 12-lead ECG, and transthoracic echocardiography (M-mode, two-dimensional and Doppler, Vivid 7, GE Medical). The echocardiographic examinations were carried out by experienced cardiologists. Cine MRI was performed with a 1.5 T system (Sonata, Siemens Medical Solution) and a body array coil. A retrospectively ECG-gated segmented Steady State Free Precession imaging was used with following parameters: echo time 1.6 ms, repetition time 3.0 ms, matrix 256×256, field of view 240×340 mm, flip angle 52 degree. Short-axis cine stack and a long-axis cine slice of both ventricles were obtained with a section thickness 6 mm, intersection gap 20%, and temporal resolution 42–49 ms.

### Image Analysis

The left and right ventricles and epicardium were semi-automatically segmented by a technician together with a radiologist from each time frame of the cine MRI series with a software tool developed for this purpose [18]. The automatic segmentation took 1–2 minutes. To reach the optimal segmentation accuracy the time used for the manual refinement was not limited. In the tool, an *a priori* heart model, consisting of triangulated surfaces of the ventricles and epicardium, was deformed to fit both short- and long-axis MRI data. Because the same *a priori* model was used for each subject, the number of the surface points was identical in each case (906 points for left ventricle, 1516 for right ventricle, and 906 for epicardium), and the point-correspondence existed between all the subjects and time frames. The surfaces were rigidly aligned in the same coordinate system to remove the position and orientation variations from the data. This enabled point-wise comparison of the cardiac motion in the study population. The heart surface models were also used to derive 86 MRI parameters that describe anatomical and physiological features of the heart, as described earlier [9].

### Lipidomics

The lipidome was analyzed as described previously [19]. In brief, serum samples (10 µl) diluted with 0.15 M NaCl (10 µl) and spiked with a standard mixture containing 10 lipid species were extracted with a mixture of chloroform and methanol 2∶1 (100 µl). The extraction time was 0.5 h and the lower organic phase was separated by centrifuging at 10 000 r.p.m. for 3 min. Another standard mixture containing three labeled lipid species was added to the extracts and the lipids were analyzed on a Waters Q-Tof Premier mass spectrometer combined with an Acquity Ultra Performance LC™ (UPLC). The column, kept at 50°C, was an Acquity UPLC™ BEH C18 1×50 mm with 1.7 µm particles. The solvent system included water (1% 1 M ammonium acetate, 0.1% HCOOH) and a mixture of acetonitrile and 2-propanol (5∶2, 1% 1 M NH_4_Ac, 0.1% HCOOH). The flow rate was 0.2 ml/min and the total run time including column re-equilibration was 18 min. Data were processed using MZmine software version 0.6 [20], [21]. Lipids were identified using the internal spectral library.

Calibration was performed as follows: all monoacyl lipids except cholesterol esters, such as monoacylglycerols and monoacyl-glycerophospholipids, were calibrated with lysophosphatidylcholine PC(17∶0/0∶0) (Avanti Polar Lipids, Alabaster, AL) as an internal standard. All diacyl lipids except phosphatidylethanolamines were calibrated with phosphatidylcholine PC(17∶0/17∶0) (Avanti Polar Lipids), the phosphatidylethanolamines with PE(17∶0/17∶0) (Avanti Polar Lipids) and the triglycerides and cholesterol esters with triglyceride TG(17∶0/17∶0/17∶0) (Larodan Fine Chemicals, Malmö, Sweden).

### Statistical analyses of variable dependencies

We calculated the marginal correlations the Pearson correlations, which indicate the degree of linear dependency between a pair of variables. The partial correlations were calculated as the Pearson correlations, with the effect of a set of control variables removed. In case the set of control variables includes all other variables the partial correlation is “full” and it depicts the linear dependency between the pair of variables that cannot be explained by any linear combination of the other variables. In “omics” applications where the number of variables is usually much larger than the number of samples, like in our case here, it is not feasible to calculate the full partial correlations, but efficient algorithms have been developed to estimate them with restricted, *q*-order, partial correlations in which a set of *q* variables are controlled [13]. In the network presentation we used here, the width of a link is proportional to the inverse of the non-rejection rate, which indicates the confidence with which the hypothesis of null partial correlation is not rejected, that is, the chance of the edge not existing in the graph is the smaller the smaller the non-rejection rate is. All calculations were done in R using the package *qpgraph* and networks were visualized using Cytoscape (www.cytoscape.org) and yEd (www.yworks.com) graphical editors.

### Regression of lipid profiles on MRI parameters

In addition to correlation based analysis, regression methods were applied to elucidate dependencies between the MR image parameters and the lipid profiles in more detail. Due to the large number of variables compared to sample size the regression must be regularized and we chose to apply the elasticnet regression method [22] as implemented in the *elasticnet* package of R. Elasticnet is convenient for our current application because the L_1_ and L_2_ penalties on the regression coefficients can be flexibly tuned by changing *u_1_* and *u_2_* respectively in Equation (1), which allows exploration of solutions ranging from simple models with no lipids at all to more complex ones that are either sparse, containing non-correlated lipids, or non-sparse with several correlated lipids included. Elasticnet solution is obtained by minimizing
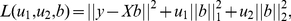
(1)where *u*_1_ and *u*_2_ are tuning parameters, *y* is the response variable and *X* a matrix containing the explanatory variables. In this study, each of the image parameters were separately explained by the whole lipid profile data and the tuning parameters were selected by minimizing the mean cross-validation error:
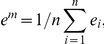


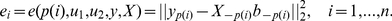
(2)Above *p = (p(1), …., p(n))* is partition of the samples into *n* blocks and *−p(i)* refers to all samples but those that belong to the *i-*th block (which belong to *p(i)*). Here we randomly assigned the samples into *n = 5* blocks for 100 times and calculated the average errors.

The cross-validation errors were also used to assess whether the lipid profiles in general explained an image parameter. The criterion was defined as follows:
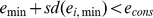
(3)


→ The image parameter in question can be explained with the lipid profiles.

Above *e*_min_ is the minimum average cross-validation error, *e*_i,min_ is the standard deviation of the cross-validation errors over the *n* blocks for the model that gives the minimum average error and *e*_cons_ is the average cross-validation error of the constant model. In other words, if the minimum mean cross-validation error plus one standard deviation of the error at minimum was lower than the mean cross-validation error of the constant model, *i.e.*, a model that contained no lipids, then the image parameter was interpreted to be dependent on the lipid profiles. The final model for each image parameter was estimated using all samples and the pair of tuning parameters that corresponded to the minimum average cross validation error.

### Association of lipid values and heart shape

A mean heart surface model, 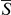, was computed from the heart surface models of Group 2. The heart model consisted both end-diastole and end-systole phases. In addition, normal vectors 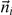 were computed for each surface point *i* of the mean heart model.

For each sample subject in Groups 1&2, the heart surface model's deviation from the mean heart model along the surface normal was computed:
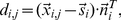
(4)where 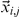 is the coordinates of *i-*th surface points of sample subject *j* and 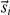 the coordinates of mean heart surface model.

A linear regression model was computed for the values of a lipid and the shape parameters 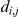 for each surface point:
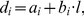
(5)where 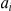 and 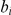 are regression weights solved from the sample subject data. The mean lipid value was computed for Group 2, 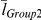, and the new shape parameters, 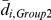, were computed from Equation 5 for the mean lipid value. The heart shape model corresponding to the mean lipid value of Group 2 was obtained from
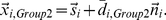
(6)Then, the mean lipid value was changed by two standard deviations according to the difference observed between Groups 1 and 2. The heart surface model corresponding to the new lipid concentration was computed as described above for the mean lipid value.

From the surface models observed, wall thickness along the surface normal was determined for each surface point of left ventricle for end-diastole and end-systole, and their difference was used as the wall thickening measure. Amount of wall motion along surface normal was computed from the location of a surface point in end-diastole and end-systole.

## Supporting Information

Table S1 Comparisons of mean values of lipid variables in Group 1 *vs.* Group 2, and Group 3 *vs.* Group 4, respectively (two-sided *t*-test). Reported fold change is the median of Group 1 or Group 3 divided by the median of Group 2 or Group 4, respectively.
(PDF)
