# Supplementary material for: Serum Lipidomics Meets Cardiac Magnetic Resonance Imaging: Profiling of Subjects at Risk of Dilated Cardiomyopathy
Source: PLoS One. 2011 Jan 20;6(1):e15744. doi: 10.1371/journal.pone.0015744 (PMC3024392; doi:10.1371/journal.pone.0015744)
Supplement: Table S1 — Comparisons of mean values of lipid variables in Group 1 vs. Group 2, and Group 3 vs. Group 4, respectively (two-sided t-test). Reported fold change is the median of Group 1 or Group 3 divided by the median of Group 2 or Group 4, respectively. (PDF) [file pone.0015744.s001.pdf]

Table S1. Comparisons of mean values of lipid variables in Group 1 vs. Group 2, and Group 3 vs. Group 4, respectively (two-sided *t*-test). Reported fold change is the median of Group 1 or Group 3 divided by the median of Group 2 or Group 4, respectively.

	**Lamin+** Group 1 *vs.* Group 2		**Lamin-** Group 3 *vs.* Group 4	
**Lipid variable**	***P*-value**	**Fold**	***P*-value**	**Fold**
TG(51:3)/TG(51:2)	0.0002	-0.45	0.1605	-0.29
DG(44:7)	0.0004	-0.26	0.1304	-0.24
TG(49:3)	0.0006	-0.99	0.0104	-0.79
PE(40:6)	0.0014	-1.23	0.5737	-0.33
PE(38:5)	0.0019	-1.08	0.1275	-0.47
TG(58:7)/TG(58:6)	0.0024	-0.55	0.9591	-0.01
TG(53:4)/TG(53:3)	0.0041	-0.33	0.3823	-0.26
TG(56:8)/TG(56:7)	0.0041	-0.55	0.5737	-0.14
PE(36:1)	0.0052	-0.52	0.4418	0.24
PE(40:6)	0.0052	-0.88	0.8785	0.12
PS(38:2)	0.0052	-0.82	0.0499	-0.52
TG(56:4)/TG(56:3)	0.0052	-0.54	0.0379	-0.29
PS(38:1)	0.0066	-1.04	0.6454	-0.03
PE(34:1)	0.0083	-0.69	0.9591	-0.13
PE(36:3)	0.0083	-0.49	0.5737	-0.38
PE(38:3)	0.0083	-0.55	0.3823	-0.36
PS(38:0)	0.0086	-0.91	0.3823	0.31
PE(40:4)	0.0104	-0.47	1.0000	-0.08
PS(38:1)	0.0104	-1.06	0.9591	-0.28
PS(40:0)	0.0104	-0.59	0.7525	0.00
TG(48:4)	0.0104	-0.58	0.8785	-0.03
TG(53:3)/TG(51:3)	0.0104	0.36	0.9591	-0.13
PC(40:8)/PC(40:7).2	0.0128	0.59	0.8785	-0.01
PE(36:2)	0.0128	-0.68	0.6454	0.16
PE(41:8)	0.0128	-0.95	0.1049	0.57
PE(34:0)	0.0151	-0.23	0.3282	0.47
PS(38:0)	0.0151	-0.90	0.1949	-0.24
PC(40:5)/PC(40:4)	0.0158	0.59	0.3282	0.67
PE(40:6)/PE(38:6e)	0.0158	-0.40	0.6454	-0.03
PE(40:7)/PE(38:7e)	0.0158	-0.28	0.6454	0.11
PS(32:5e)	0.0158	-0.65	0.7209	0.16
PS(38:1)	0.0158	-0.97	0.9591	-0.28
PS(40:0)	0.0158	-0.69	0.7525	-0.09
PS(40:0)/PS(38:0).1	0.0158	0.15	0.3823	0.21
TG(49:2)/TG(49:1)	0.0158	-0.23	0.0030	-0.79
TG(50:10)	0.0158	-0.64	0.0148	-0.76
PE(26:5)	0.0192	-0.76	0.3282	0.18

PE(38:4)	0.0192	-0.54	0.5737	-0.17
PE(38:6)	0.0192	-0.65	0.7984	-0.01
PE(40:4)	0.0192	-0.56	0.2345	-0.36
PE(26:5)	0.0233	-0.76	0.5054	0.13
PE(38:5e)	0.0233	-0.69	0.8785	-0.05
PE(40:6)	0.0233	-1.12	0.7984	-0.03
TG(54:8)/TG(54:7).1	0.0233	-0.31	0.0650	-0.80
PE(38:4)	0.0278	-0.51	0.5054	-0.44
PC(38:4)/PC(38:3)	0.0281	-0.40	0.2345	-0.37
PE(38:4)	0.0281	-0.80	0.4418	-0.44
PE(38:6e)	0.0281	-0.60	0.1605	0.18
PS(36:1)	0.0281	-1.01	0.1304	0.36
PE(27:5)	0.0336	-0.64	0.1605	-0.40
PE(27:5)	0.0336	-0.64	0.1605	-0.44
PE(38:2)	0.0336	-0.56	0.7984	0.11
PE(38:6)	0.0336	-0.84	0.7209	-0.02
PE(40:7)	0.0336	-1.10	1.0000	0.15
TG(50:2)/TG(50:1)	0.0336	-1.00	0.0030	-3.22
TG(58:7)	0.0336	-0.82	0.3282	0.43
PE(27:5)	0.0385	-0.89	0.1049	0.45
PE(38:7e)	0.0385	-0.68	0.9581	-0.05
SM(d18:1/17:1)	0.0408	-0.26	0.8334	-0.08
PE(35:5e)	0.0451	-0.58	0.2933	0.25
TG(56:5)	0.0451	-1.23	0.3282	0.37
PC(38:4)	0.0473	-0.36	1.0000	0.04
PS(38:1)	0.0473	-0.61	0.0650	-0.73
TG(49:1)/TG(47:1)	0.0473	0.30	0.3823	0.07
TG(51:2)/TG(49:2)	0.0473	0.35	0.1049	0.35
TG(52:6)/TG(52:5)	0.0473	-0.26	0.7984	0.00
TG(58:8)/TG(58:7).2	0.0473	-0.73	0.7209	0.63
PE(27:5)	0.0557	-0.85	0.1605	0.36
PE(36:0)	0.0557	-0.46	0.4418	0.21
PE(38:6)	0.0557	-0.81	0.7984	0.19
PS(34:6)	0.0557	-0.68	0.7984	-0.04
PE(40:6e)	0.0565	-0.49	0.2352	-2.81
PC(38:5e)	0.0568	-0.51	0.0356	-0.53
PC(38:5e)	0.0568	-0.11	0.0830	-0.18
LysoPC(22:6)	0.0612	-0.50	0.2345	0.37
PE(26:6)	0.0652	-0.42	0.2345	-0.34
PE(36:2)	0.0652	-0.58	0.6454	0.27
PE(38:5)	0.0652	-0.99	0.1033	-0.57
PS(38:3e)	0.0652	-0.67	0.8785	0.06
PS(40:7e)	0.0652	-0.88	0.3282	-0.28
SM(d18:1/16:1)/SM(d18:1/16:0)	0.0652	-0.15	0.1049	0.25
TG(58:8)	0.0652	-1.73	0.5054	0.36
PC(34:4)/PC(34:3)	0.0759	-0.51	0.6454	-0.18

PC(34:5)/PC(34:4).1	0.0759	-0.36	0.7984	-0.10
PC(36:4)	0.0759	-0.24	0.7984	-0.15
PC(36:5)	0.0759	-0.56	0.1049	0.46
PC(40:7)	0.0759	-0.34	0.1049	-0.43
PC(40:7)/PC(38:7)	0.0759	-0.91	0.2345	-0.17
PE(38:1)	0.0759	-0.41	0.3282	0.25
PE(38:3)/PE(36:3).1	0.0759	0.49	0.2345	0.34
PG(38:4)/PG(36:4)	0.0759	-0.31	0.7209	0.04
PS(41:6)	0.0759	-0.38	0.8785	0.09
TG(53:3)/TG(53:2)	0.0759	-0.29	0.0379	-0.18
TG(42:0)	0.0760	-1.39	0.7209	-0.05
PE(36:3)	0.0761	-0.33	0.2786	-0.13
PC(27:5)	0.0874	0.48	0.0146	-0.78
Cer(d18:1/24:1)/Cer(d18:1/24:0)	0.0879	-0.26	0.3282	-0.24
PA(41:8e)	0.0879	0.67	0.0830	-0.32
PE(38:5)/PE(38:4)	0.0879	-0.81	0.5054	-0.31
PE(40:4)	0.0879	-0.35	0.5737	-0.24
PE(40:4)/PE(40:3)	0.0879	-1.04	0.4418	0.33
PE(40:6)/PE(38:6).1	0.0879	-0.22	0.7209	0.07
PI(38:4)/PI(38:3)	0.0879	-0.25	0.0830	0.21
PS(27:5)	0.0879	-0.44	0.9591	0.01
TG(51:1)/TG(49:1)	0.0879	0.28	0.6454	0.16
TG(56:5)	0.0879	-0.99	0.0830	-0.65
TG(56:6)	0.0879	-1.08	0.1949	0.60
TG(58:10)	0.0879	-1.13	0.5737	0.05
PC(32:1)/PC(32:0)	0.1006	0.32	0.2345	0.34
PA(27:1)	0.1014	-0.37	1.0000	0.05
PC(40:5)/PC(38:5e)	0.1014	0.73	0.1412	0.82
PE(27:5)	0.1014	-0.57	0.1889	-0.59
PE(40:7e)	0.1014	-0.48	0.7130	-0.14
TG(58:8)/TG(58:7).1	0.1014	-0.49	0.5737	0.13
TG(58:9)	0.1014	-1.38	0.7209	0.16
PE(38:3)	0.1148	-0.26	0.4619	0.17
PS(27:6)	0.1149	-0.28	0.7209	-0.23
PC(40:8)/PC(40:7)	0.1164	0.96	0.9591	-0.08
TG(54:8)	0.1164	-0.94	0.4418	0.24
PC(34:2)/PC(34:1)	0.1330	-0.22	0.4418	-0.21
PE(25:5)	0.1330	-0.21	0.5054	-0.28
PS(41:7)	0.1330	-0.55	0.1605	-0.55
TG(50:1)/TG(48:1)	0.1330	0.41	0.3282	-0.16
TG(56:8)	0.1330	-0.62	0.3282	0.58
TG(58:7)	0.1330	-0.45	0.3181	0.45
DG(50:2e)	0.1394	-0.75	0.8334	-0.12
PE(40:8e)	0.1394	-0.33	1.0000	-0.13
TG(44:0)/TG(42:0)	0.1394	0.81	0.3823	0.88
LysoPC(20:3)	0.1513	-0.24	0.9163	-0.19

PC(25:4)	0.1513	-0.44	0.2698	0.08
PC(32:2)/PC(32:1)	0.1513	-0.21	0.4418	-0.19
PC(36:4)/PC(34:4)	0.1513	0.23	0.1605	-0.71
PC(38:5)/PC(38:4)	0.1513	0.11	0.4418	0.11
PC(40:5)/PC(38:5).1	0.1513	0.32	0.3282	0.93
PC(40:7)/PC(40:6)	0.1513	-0.84	0.0650	-0.53
PE(34:1)/PE(34:0)	0.1513	-0.18	0.6454	-0.14
PE(34:4e)	0.1513	-0.41	0.2345	-0.29
PE(36:3e)	0.1513	-0.51	0.6454	0.01
PE(40:3)/PE(38:3).2	0.1513	0.74	0.6454	-0.27
PS(40:0)/PS(38:0)	0.1513	0.24	0.3823	-0.36
TG(50:1)	0.1513	0.82	0.0379	1.93
TG(50:3)/TG(50:2).1	0.1513	-0.19	0.3282	-0.14
TG(52:3)/TG(52:2)	0.1513	-0.06	0.3823	-0.07
TG(54:6)/TG(54:5).1	0.1513	-0.34	0.8785	0.11
TG(54:7)/TG(54:6)	0.1513	-0.36	0.5737	0.01
TG(54:7)/TG(54:6).1	0.1513	-0.36	0.5054	0.41
TG(56:8)	0.1513	-0.54	0.3282	0.58
PA(34:0)	0.1567	0.07	0.1304	-0.13
PC(40:6)	0.1579	-0.49	0.1605	0.35
TG(43:6)	0.1579	-0.78	0.1033	0.50
PC(40:7)	0.1713	-0.94	0.1605	-0.47
PC(40:7)/PC(38:7).2	0.1713	-0.34	0.5737	0.17
PE(36:1)	0.1713	-0.55	0.1605	0.45
PE(38:5)	0.1713	-0.56	0.0207	-0.66
PE(38:5)/PE(38:4).1	0.1713	-0.58	0.6454	-0.31
TG(42:1)/TG(42:0)	0.1713	1.15	0.4418	1.16
TG(46:1)/TG(46:0)	0.1713	0.34	0.8785	-0.13
TG(54:8)/TG(54:7)	0.1713	-0.22	0.4418	-0.51
TG(56:5)/TG(54:5).1	0.1713	-0.82	0.0030	-0.81
TG(56:9)	0.1713	-0.90	0.1719	0.24
TG(58:10)/TG(58:9)	0.1713	-0.20	0.8785	0.00
PC(36:6)	0.1780	-0.45	0.3435	0.11
PC(27:5)	0.1781	-0.39	1.0000	-0.02
LysoPC(20:4)	0.1890	-0.27	0.8785	-0.03
TG(54:7)	0.1890	-0.53	0.1049	0.76
PC(38:4)/PC(36:4)	0.1932	-0.43	0.4418	0.58
PC(38:6)	0.1932	-0.43	0.5737	0.03
PC(40:7)/PC(38:7).1	0.1932	-0.70	0.5737	0.17
PC(40:8)	0.1932	-0.79	0.1605	-0.22
PE(27:5)/PE(25:5)	0.1932	-0.14	0.4418	-0.11
PE(36:3)/PE(36:2).1	0.1932	0.25	0.3823	-0.32
PE(40:4)/PE(38:4)	0.1932	-0.44	0.6454	0.22
PE(40:6)/PE(38:6)	0.1932	-0.16	0.7984	0.04
PS(30:0)	0.1932	-0.29	0.1304	-0.22
PS(40:7e)	0.1932	-0.30	0.3282	-0.28

TG(42:1)/TG(42:0).1	0.1932	0.29	0.1304	0.80
TG(44:0)/TG(42:0).1	0.1932	0.04	0.0379	0.24
TG(48:3)/TG(48:2)	0.1932	-0.34	1.0000	-0.05
TG(53:5)/TG(53:4)	0.1932	-0.13	0.1049	-0.49
TG(54:2)/TG(52:2)	0.1932	0.55	0.0047	0.77
TG(54:3)/TG(54:2)	0.1932	-0.12	0.0011	-1.33
TG(56:7)	0.1932	-1.11	0.2786	0.70
TG(56:7)/TG(54:7)	0.1932	0.26	1.0000	0.13
PE(38:2)	0.2003	-0.18	0.5737	-0.03
SM(d18:1/17:0)	0.2116	-0.20	1.0000	0.12
PC(34:4)	0.2119	-0.25	0.8746	-0.05
PG(38:4)	0.2120	-0.22	0.3823	0.11
PC(34:5)/PC(34:4)	0.2169	0.26	0.4418	0.17
PC(36:3)/PC(36:2)	0.2169	0.15	0.1949	0.10
PE(38:3)	0.2169	-0.42	0.9591	0.12
PE(38:6)/PE(38:5).2	0.2169	0.25	0.2786	0.51
SM(d18:1/24:3)/SM(d18:1/24:2)	0.2169	-0.08	0.3282	0.39
TG(50:0)/TG(48:0)	0.2169	0.14	0.7209	0.20
TG(52:2)/TG(50:2)	0.2169	0.71	0.0148	1.81
TG(56:4)/TG(54:4)	0.2169	-0.58	0.4418	0.02
TG(56:4)/TG(54:4).1	0.2169	-0.58	0.4418	0.02
PC(28:2)	0.2234	-0.25	0.2933	0.31
SM(d18:1/25:4)	0.2366	-0.30	0.7209	0.26
LysoPC(16:0)	0.2426	-0.20	0.5737	0.09
LysoPC(20:4)/LysoPC(20:3).1	0.2426	0.26	0.9591	0.18
PC(36:4)/PC(34:4).1	0.2426	-0.21	0.0499	-0.84
PC(38:6)	0.2426	-0.41	0.4418	0.30
PC(40:5)/PC(38:5e).1	0.2426	0.46	0.1304	0.41
PE(36:1)/PE(34:1)	0.2426	0.19	0.3282	0.36
PE(40:6)/PE(38:6).2	0.2426	-0.10	0.9591	-0.02
PE(40:7)/PE(40:6).2	0.2426	-0.14	0.5737	0.10
PS(38:2)/PS(38:1).3	0.2426	-0.05	0.5737	-0.06
SM(d18:1/16:1)	0.2426	-0.09	0.1605	0.40
SM(d18:1/18:0)	0.2426	-0.14	0.6454	-0.09
SM(d18:1/23:1)/SM(d18:1/23:0)	0.2426	0.09	0.9591	-0.06
TG(46:1)/TG(44:1)	0.2426	0.49	0.5737	-0.11
TG(51:3)/TG(49:3)	0.2426	0.43	0.0281	1.98
TG(56:5)/TG(54:5)	0.2426	-0.26	0.0830	-1.26
TG(58:7)	0.2426	-0.65	0.3282	0.52
PI(38:1)	0.2633	-0.18	0.3141	-0.04
SM(d18:1/17:1)/SM(d18:1/17:0)	0.2638	-0.10	0.6454	0.03
TG(52:6)	0.2642	-0.48	0.1049	0.45
LysoPC(18:0)	0.2703	-0.27	0.8785	-0.13
PC(32:0)	0.2703	-0.20	0.1605	0.22
PC(38:5)/PC(36:5).1	0.2703	0.42	0.0830	-0.57
PE(27:5)	0.2703	-0.24	0.8785	-0.05

PE(40:3)/PE(38:3).1	0.2703	0.82	0.0650	-0.68
TG(46:2)/TG(46:1)	0.2703	-0.14	0.2786	-0.22
TG(52:2)/TG(52:1)	0.2703	-0.15	0.0104	-1.57
TG(53:2)/TG(51:2)	0.2703	0.17	0.6454	0.09
TG(58:7)/TG(56:7)	0.2703	-0.38	0.2345	-0.50
TG(50:2)	0.2933	0.06	0.0054	-1.05
PC(38:7)	0.3000	-0.26	0.0379	-0.46
PC(40:4)	0.3000	-0.30	0.1949	-0.27
SM(d18:1/24:3)	0.3000	-0.21	0.1605	0.48
TG(48:1)/TG(48:0)	0.3000	0.42	0.5054	-0.19
TG(49:3)/TG(49:2)	0.3000	-0.66	0.0379	-2.04
TG(50:5)/TG(50:4)	0.3000	-0.09	0.0207	-0.40
TG(54:6)/TG(52:6)	0.3000	0.17	0.8785	-0.27
TG(56:5)/TG(56:4).1	0.3000	-0.73	0.0499	-1.07
TG(56:7)/TG(54:7).1	0.3000	0.28	0.9591	0.06
TG(54:1)	0.3086	0.53	0.0499	2.60
Cer(d18:1/24:0)/Cer(d18:1/22:0)	0.3316	0.08	0.3282	0.16
LysoPC(20:5)/LysoPC(20:4).1	0.3316	0.32	0.8785	0.16
PC(32:5)	0.3316	-0.08	0.2470	0.58
PC(38:5)	0.3316	-0.38	0.3823	0.06
PC(40:5)/PC(38:5)	0.3316	0.09	0.7984	0.09
PE(38:6)/PE(38:5)	0.3316	0.82	0.1949	0.51
PE(40:3)/PE(38:3)	0.3316	0.59	0.0148	-0.61
TG(50:2)/TG(48:2)	0.3316	-1.12	0.0070	-2.86
TG(52:1)/TG(52:0)	0.3316	0.11	0.3282	0.28
TG(54:2)	0.3316	0.30	0.0281	2.02
TG(58:8)/TG(58:7)	0.3316	-0.39	0.8785	0.52
LysoPC(18:0)	0.3408	-0.20	0.9163	0.01
TG(56:2)	0.3408	0.24	0.0047	1.61
PC(40:7)	0.3409	-0.34	0.2786	0.12
PG(36:2)/PG(34:2)	0.3409	-0.17	0.5737	-0.24
SM(d18:1/18:1)	0.3577	-0.06	1.0000	0.17
PC(38:7)/PC(38:6)	0.3653	0.69	0.5737	-0.27
PE(38:3)/PE(38:2).1	0.3653	0.07	0.7209	-0.14
TG(48:2)/TG(48:1)	0.3653	-0.15	0.0070	-0.82
TG(54:7)	0.3653	-0.74	0.1049	1.12
TG(58:8)/TG(56:8).1	0.3653	-0.08	0.2786	-0.21
Cer(d18:1/17:0)	0.3752	0.03	0.2345	-0.13
PA(27:1)	0.3752	-0.06	0.1889	-0.32
PC(26:4)	0.3930	-0.11	0.0830	-0.61
Cer(d18:1/24:1)/Cer(d18:1/24:0).1	0.4009	-0.44	0.8785	0.05
LysoPA(25:5)	0.4009	0.55	0.1949	-0.14
PC(38:3e)	0.4009	0.49	0.2345	-0.26
PC(40:7)/PC(40:6).1	0.4009	-0.58	0.0207	-1.02
PE(38:5)/PE(38:4).3	0.4009	-0.52	0.1949	-0.32
PE(40:6e)	0.4009	-0.49	0.2345	0.39

PS(38:1)/PS(38:0).1	0.4009	-0.29	0.1049	0.31
SM(d18:1/18:3)	0.4009	-0.09	0.7209	-0.02
SM(d18:1/24:4)/SM(d18:1/24:3)	0.4009	0.13	0.1605	-0.32
TG(52:1)	0.4009	0.17	0.0499	2.53
TG(53:5)	0.4009	-0.66	0.2072	0.32
TG(56:6)/TG(54:6).1	0.4009	-0.17	0.9591	-0.08
TG(56:6)/TG(56:5)	0.4009	0.19	0.0047	1.08
TG(56:6)/TG(56:5).1	0.4009	0.07	0.3282	0.07
TG(58:6)/TG(56:6)	0.4009	0.15	0.5054	-0.18
SM(d18:1/18:2)	0.4116	-0.22	0.5271	-0.07
SM(d18:1/26:5)	0.4116	-0.37	0.3282	-0.17
TG(47:1)	0.4116	-0.48	0.1605	1.45
MG(20:1)	0.4306	0.12	0.2933	-0.09
Cer(d18:1/25:1)	0.4385	-0.31	0.2933	0.41
PA(41:7)	0.4385	0.29	0.0281	-0.17
PC(36:6)/PC(36:5)	0.4385	-0.33	0.8785	0.03
PC(36:6)/PC(36:5).1	0.4385	0.20	0.3823	-0.14
PC(38:7)/PC(38:6).2	0.4385	0.51	0.1304	-0.55
PC(40:6)/PC(38:6).2	0.4385	0.34	0.7984	-0.06
PC(40:6)/PC(40:5)	0.4385	-0.16	0.5054	-0.11
PC(40:6)/PC(40:5).1	0.4385	0.29	0.2345	-0.10
PE(36:3)/PE(36:2)	0.4385	0.28	0.3282	-0.47
SM(d18:1/18:2)/SM(d18:1/18:1)	0.4385	0.09	0.2345	-0.24
SM(d18:1/26:5)/SM(d18:1/26:4)	0.4385	-0.11	0.0650	-0.18
TG(48:1)	0.4385	0.51	0.0148	2.35
TG(50:3)/TG(50:2)	0.4385	0.35	0.0207	1.96
TG(52:5)/TG(50:5)	0.4385	0.30	0.4418	0.43
TG(54:3)/TG(52:3)	0.4385	-0.02	0.7209	-0.12
PA(34:0)	0.4779	0.08	0.9591	0.09
PC(38:7)	0.4779	-0.40	0.0379	-0.46
PE(38:4)	0.4779	-0.30	0.6454	-0.10
PS(38:2)/PS(38:1)	0.4779	0.55	0.0499	-0.47
SM(d18:1/14:0)	0.4779	-0.15	0.4619	0.21
SM(d18:1/15:0)	0.4779	-0.10	0.7984	0.14
SM(d18:1/25:1)	0.4779	-0.12	0.8785	0.02
TG(44:1)/TG(42:1)	0.4779	-0.08	0.8785	-0.39
TG(52:5)	0.4779	-0.12	0.1605	0.77
TG(54:5)	0.4779	-0.38	0.3282	0.60
TG(56:4)	0.4779	-0.07	0.2786	0.72
TG(56:6)/TG(54:6)	0.4779	-0.16	0.9591	0.07
TG(56:7)/TG(56:6)	0.4779	0.30	0.4418	0.49
TG(58:9)/TG(58:8)	0.4779	-0.11	0.0379	-0.19
Cer(d18:1/24:0)	0.4904	0.09	0.1889	0.47
PC(34:5)	0.4904	-0.30	0.4302	0.10
TG(53:4)	0.4904	-0.49	0.1049	0.65
PC(32:5)	0.5112	-0.09	0.5992	-0.07

PA(38:7e)	0.5113	0.19	0.0207	-0.21
SM(d18:0/20:4)	0.5113	-0.42	0.0927	-0.29
LysoPC(18:1)/LysoPC(18:0).1	0.5190	0.08	0.4418	-0.22
PC(36:2)/PC(34:2)	0.5190	-0.08	0.7209	0.07
PC(36:5)/PC(36:4).1	0.5190	0.21	0.5054	0.31
PC(40:6)/PC(38:6)	0.5190	0.28	0.7984	-0.15
PE(38:2)/PE(36:2)	0.5190	0.12	0.8785	-0.18
PS(38:1)/PS(36:1)	0.5190	-0.15	0.4418	-0.26
PS(38:1)/PS(38:0)	0.5190	0.01	0.5737	-0.17
PS(38:2)/PS(38:1).1	0.5190	0.15	0.1049	-0.32
SM(d18:1/16:0)	0.5190	-0.18	0.7209	0.25
TG(48:4)/TG(48:3)	0.5190	-0.63	0.3282	-0.98
TG(50:1)/TG(50:0)	0.5190	0.45	0.1949	-0.60
TG(51:3)	0.5190	-0.24	0.1049	0.60
TG(54:4)/TG(54:3)	0.5190	-0.10	0.5737	-0.05
TG(54:6)	0.5190	-0.22	0.1049	0.64
TG(56:3)/TG(56:2)	0.5190	-0.08	0.0070	-0.59
TG(56:7)	0.5190	-0.37	0.1605	0.40
TG(56:8)/TG(56:7).1	0.5190	-0.07	0.8785	0.10
TG(51:1)	0.5326	0.05	0.0281	2.04
PG(40:3e)	0.5543	0.22	0.2072	0.60
LysoPC(18:2)	0.5619	-0.13	0.2072	-0.26
LysoPC(18:3)	0.5619	-0.08	0.1605	0.27
PC(34:5)/PC(32:5)	0.5619	-0.12	0.5054	0.15
PC(36:2)/PC(36:1)	0.5619	-0.05	0.3282	-0.43
PC(36:5)/PC(36:4)	0.5619	-0.10	0.5737	0.03
PC(38:2)/PC(36:2)	0.5619	-0.01	0.1304	0.49
PC(38:5)	0.5619	-0.25	0.3282	-0.34
PC(38:5)/PC(36:5)	0.5619	0.08	0.7984	-0.35
PC(40:4e)	0.5619	0.54	0.2345	-0.36
PE(38:3)/PE(38:2)	0.5619	0.03	0.8785	-0.23
PE(40:4)/PE(38:4).1	0.5619	-0.16	0.4418	0.26
SM(d18:1/18:1)/SM(d18:1/18:0)	0.5619	0.01	0.7984	0.08
SM(d18:1/26:4)	0.5619	-0.21	0.3282	0.22
TG(50:5)	0.5619	-0.22	0.1949	0.38
TG(51:2)/TG(51:1)	0.5619	-0.13	0.0379	-0.40
TG(51:4)	0.5619	-0.11	0.3282	0.31
TG(53:2)	0.5619	0.25	0.0379	1.28
TG(54:2)/TG(54:1)	0.5619	-0.26	0.1605	-0.46
TG(54:6)	0.5619	-0.22	0.2345	0.40
TG(56:3)/TG(54:3)	0.5619	0.10	0.1049	0.34
TG(56:5)/TG(56:4)	0.5619	-0.56	0.0281	-1.63
Cer(d18:1/24:1)	0.5765	-0.17	0.3823	0.41
TG(50:0)	0.5766	0.14	0.0281	2.85
PC(40:5e)	0.5992	0.02	0.2268	-0.14
SM(d18:1/23:0)	0.5993	0.02	0.5054	0.19

Cer(d18:1/22:6)	0.6063	-0.18	0.1605	-0.26
Cer(d18:1/23:2)	0.6063	0.14	0.8785	0.06
LysoPC(18:1)	0.6063	-0.05	0.6454	0.01
LysoPC(18:2)/LysoPC(18:1)	0.6063	0.23	0.3282	-0.35
PC(34:1)/PC(32:1)	0.6063	-0.21	0.0650	-0.39
PC(36:2)	0.6063	-0.20	0.9591	-0.06
PC(40:5)	0.6063	0.12	0.0519	0.43
PE(38:1)/PE(36:1).1	0.6063	-0.03	0.3282	0.32
PE(38:2)/PE(36:2).1	0.6063	-0.10	0.8785	-0.05
PE(38:3)/PE(36:3e)	0.6063	-0.13	0.7209	-0.09
PE(38:5)/PE(38:4).2	0.6063	-0.06	0.0830	-0.33
SM(d18:1/24:2)	0.6063	-0.26	0.8785	0.18
TG(46:2)/TG(44:2)	0.6063	0.11	0.7209	-0.11
TG(49:2)	0.6063	-0.32	0.1304	0.68
TG(52:4)/TG(50:4)	0.6063	0.18	0.0030	-0.49
TG(52:5)/TG(52:4)	0.6063	-0.16	0.1304	0.50
TG(54:1)/TG(52:1)	0.6063	0.06	0.9591	-0.17
TG(56:2)/TG(54:2)	0.6063	-0.04	0.7209	-0.12
TG(58:6)	0.6063	-0.03	0.1949	0.42
PC(41:4)	0.6452	0.02	0.7984	0.01
PC(26:1e)	0.6456	-0.11	0.3282	-0.15
LysoPC(20:3)	0.6457	-0.06	0.3282	-0.18
LysoPC(18:3)/LysoPC(18:2)	0.6522	-0.09	0.0650	0.49
LysoPE(23:3e)	0.6522	0.02	0.4606	-0.18
PC(32:1)	0.6522	0.36	0.3282	0.34
PC(36:3)/PC(34:3)	0.6522	0.04	0.6454	0.01
PC(36:4)/PC(36:3)	0.6522	0.11	0.0650	-0.61
PC(40:6)/PC(38:6).1	0.6522	0.07	0.0207	0.41
PC(40:8)/PC(40:7).1	0.6522	0.01	0.1949	-0.32
PE(26:6)/PE(26:5)	0.6522	0.26	0.2786	-0.96
PE(36:2)/PE(36:1)	0.6522	0.04	0.2345	-0.29
PE(38:4)/PE(38:3)	0.6522	-0.04	0.3823	-0.62
PE(40:7)/PE(40:6).1	0.6522	-0.08	0.7984	-0.15
PS(38:2)/PS(38:1).2	0.6522	0.47	0.0499	-0.47
SM(d18:1/21:0)	0.6522	0.02	0.4619	0.24
TG(50:4)/TG(48:4)	0.6522	0.02	0.0830	0.95
TG(56:4)	0.6522	-0.24	0.0830	1.02
DG(28:0e)	0.6687	0.02	0.3170	-0.11
PC(41:4)	0.6692	0.02	0.8335	0.01
PC(34:4)	0.6693	0.28	0.6454	0.07
LysoPC(20:4)	0.6694	0.05	0.2473	0.23
MG(23:3e)	0.6694	0.20	0.0515	-0.26
PI(38:4)	0.6694	0.05	0.1049	-0.74
Cer(d18:1/26:1)	0.6934	-0.07	0.1412	0.44
PC(41:5)	0.6935	0.14	0.2072	-0.58
Cer(d18:1/26:1)/Cer(d18:1/24:1)	0.6994	-0.01	0.9591	0.07

PC(32:0e)	0.6994	0.13	0.1949	-0.26
PC(34:1)	0.6994	0.13	0.7984	-0.04
PE(40:7)/PE(40:6)	0.6994	-0.01	0.5737	-0.09
SM(d18:1/18:3)/SM(d18:1/18:2)	0.6994	0.05	0.5737	0.05
SM(d18:1/25:1)/SM(d18:1/23:1)	0.6994	-0.01	0.8785	-0.10
TG(48:0)/TG(46:0)	0.6994	0.10	0.3823	0.05
TG(52:0)	0.6994	0.31	0.0499	2.33
TG(52:2)/TG(50:2).1	0.6994	-0.10	0.2786	-0.09
TG(56:9)/TG(56:8).1	0.6994	-0.25	0.6454	-0.12
PC(25:4)	0.7178	0.07	0.3442	0.12
TG(56:3)	0.7179	0.24	0.0519	1.23
LysoPC(18:0)/LysoPC(16:0).1	0.7477	0.03	0.8785	-0.02
LysoPC(18:3)/LysoPC(18:2).1	0.7477	-0.11	0.1304	0.58
LysoPC(20:3)/LysoPC(18:3)	0.7477	0.00	0.1605	-0.26
PC(34:5)/PC(32:5).1	0.7477	0.28	0.6454	-0.08
PC(36:5)	0.7477	-0.22	0.7209	0.25
PC(38:6)/PC(38:5)	0.7477	0.04	1.0000	-0.05
PC(41:5)/PC(41:4).1	0.7477	0.13	0.2786	-0.61
PE(36:2)/PE(36:1).1	0.7477	-0.18	0.7209	0.34
PE(38:3)/PE(36:3)	0.7477	-0.17	0.3823	0.33
PE(40:0e)	0.7477	-0.07	0.3282	0.95
PE(40:4)/PE(38:4).3	0.7477	-0.02	1.0000	0.02
SM(d18:1/16:0)/SM(d18:1/14:0)	0.7477	0.02	0.3282	-0.16
SM(d18:1/18:0)/SM(d18:1/16:0)	0.7477	-0.07	0.3282	-0.12
SM(d18:1/23:0)/SM(d18:1/21:0)	0.7477	-0.02	1.0000	-0.04
SM(d18:1/24:1)	0.7477	-0.25	0.4418	0.15
TG(52:2)	0.7477	0.09	0.2345	0.69
TG(52:4)	0.7477	-0.22	0.3282	0.27
TG(54:6)/TG(54:5)	0.7477	0.04	0.6454	0.01
TG(58:8)/TG(56:8)	0.7477	-0.04	0.2786	-0.21
MG(25:6e)	0.7676	-0.09	0.0650	-0.41
PC(40:4)	0.7676	-0.02	0.5283	0.21
PI(38:3)	0.7676	0.31	0.1031	-0.83
TG(53:3)	0.7676	0.00	0.0830	1.10
LysoPC(18:1)/LysoPC(18:0)	0.7969	0.04	0.1605	-0.14
LysoPC(18:2)	0.7969	0.02	0.2786	-0.22
PC(34:3)/PC(34:2)	0.7969	0.25	0.1605	0.31
PC(36:1)/PC(34:1)	0.7969	-0.17	0.1605	0.24
PC(36:5)/PC(34:5)	0.7969	-0.02	0.8785	0.16
PC(38:3)	0.7969	0.15	0.1605	0.43
PC(40:4)/PC(38:4)	0.7969	0.06	0.2786	-0.60
PE(26:6)/PE(26:5).1	0.7969	-0.25	0.0830	-1.25
PE(36:1)/PE(36:0)	0.7969	0.17	0.5054	0.06
PE(38:4)/PE(38:3).2	0.7969	0.20	0.7984	0.06
SM(d18:1/18:1)/SM(d18:1/16:1)	0.7969	0.00	0.0650	-0.28
SM(d18:1/22:0)	0.7969	-0.19	0.3823	0.34

TG(44:1)/TG(44:0)	0.7969	-0.02	0.1605	0.26
TG(46:0)/TG(44:0)	0.7969	0.02	0.5737	0.04
TG(46:1)	0.7969	-0.22	0.0499	2.16
TG(48:2)	0.7969	0.42	0.1304	1.33
TG(51:4)/TG(51:3)	0.7969	-0.06	0.2345	-0.20
TG(54:4)	0.7969	-0.16	0.5737	0.37
TG(54:4)	0.7969	-0.16	0.9591	0.20
PC(40:5)	0.8182	-0.01	0.6742	0.08
DG(31:2)	0.8423	0.42	0.8785	0.01
PG(34:2)	0.8435	-0.06	1.0000	0.07
PE(30:1e)	0.8438	0.37	0.0499	-0.37
PS(40:6e)	0.8438	-0.02	0.9581	-0.06
TG(51:2)	0.8438	0.57	0.0379	1.41
Cer(d18:1/24:0)	0.8470	0.00	0.6454	0.28
PC(27:5)	0.8470	-0.07	0.5283	-0.12
PC(38:6)	0.8470	-0.01	0.5737	-0.11
PC(38:6)/PC(38:5).1	0.8470	0.05	0.3823	0.80
PE(38:1)/PE(36:1)	0.8470	0.00	0.7984	-0.05
SM(d18:1/26:4)/SM(d18:1/24:4)	0.8470	-0.20	0.5737	0.04
TG(48:0)	0.8470	-0.18	0.0281	2.47
TG(50:2)	0.8470	0.28	0.1949	0.65
TG(50:3)/TG(48:3)	0.8470	0.01	0.2345	-0.43
TG(50:4)	0.8470	-0.05	0.1304	0.76
TG(52:3)/TG(50:3)	0.8470	0.03	0.2786	-0.24
TG(54:5)	0.8470	0.16	0.2345	0.42
TG(56:8)/TG(54:8)	0.8470	0.05	0.4418	0.29
PC(27:3)	0.8692	-0.12	0.5280	0.22
SM(d18:1/21:2)	0.8693	0.00	0.4418	-0.21
LysoPE(18:4e)	0.8696	-0.20	0.0148	-0.15
PE(40:3)	0.8696	0.05	0.2786	-0.23
TG(54:3)	0.8696	-0.02	0.5054	0.66
SM(d18:1/23:3)	0.8954	0.09	0.6360	0.50
PG(36:2)	0.8954	0.00	0.8334	0.09
Cer(d18:1/23:0)	0.8955	0.09	0.3282	0.38
TG(42:1)	0.8955	0.00	0.1149	2.49
PC(38:7)	0.8955	0.06	0.6740	-0.39
SM(d18:1/24:4)	0.8955	0.06	1.0000	0.03
LysoPC(16:0)	0.8977	0.18	0.5737	0.01
LysoPC(20:5)	0.8977	-0.02	0.9591	-0.04
PC(36:1)	0.8977	-0.06	0.5737	0.14
PC(36:3)	0.8977	0.11	0.5054	0.14
PC(36:4)	0.8977	-0.13	0.1949	-0.36
PC(38:2)	0.8977	-0.07	0.1605	0.30
PE(40:4)/PE(38:4).2	0.8977	0.29	0.7209	-0.13
PS(41:7)/PS(41:6)	0.8977	-0.02	0.5737	-0.49
SM(d18:1/24:2)/SM(d18:1/24:1)	0.8977	0.01	0.6454	0.08

TG(44:1)	0.8977	-0.29	0.0499	2.25
TG(46:0)	0.8977	-0.26	0.0281	2.13
TG(48:3)	0.8977	0.19	0.0829	1.41
TG(50:3)	0.8977	-0.01	0.4418	0.25
TG(50:4)/TG(50:3)	0.8977	0.11	0.0379	0.36
TG(54:4)/TG(52:4)	0.8977	-0.16	0.7209	-0.14
TG(54:5)/TG(52:5)	0.8977	-0.09	0.2345	-0.28
TG(56:9)/TG(56:8)	0.8977	-0.14	0.6454	-0.12
TG(58:7)/TG(56:7).1	0.8977	-0.07	0.5054	-0.08
PG(36:4)	0.9215	0.10	0.4418	0.15
PC(40:6)	0.9215	-0.09	0.5737	0.14
TG(42:0)	0.9215	-0.34	0.1949	1.77
Cer(d18:1/22:0)	0.9476	-0.14	0.3282	0.22
PC(40:8)	0.9476	0.11	0.1605	-0.22
PC(40:8)	0.9476	-0.02	0.1605	-0.22
LysoPC(18:0)/LysoPC(16:0)	0.9487	-0.08	0.8785	-0.09
LysoPC(20:4)/LysoPC(20:3)	0.9487	-0.04	0.3823	0.11
LysoPC(20:5)/LysoPC(20:4)	0.9487	0.12	0.6454	-0.10
PC(34:2)/PC(32:2)	0.9487	-0.09	0.2345	-0.29
PC(38:3)/PC(38:2)	0.9487	-0.02	0.5054	-0.23
PC(38:4)/PC(36:4).1	0.9487	0.02	1.0000	0.04
PC(38:6)/PC(36:6)	0.9487	-0.06	0.9591	0.21
PC(38:7)/PC(38:6).1	0.9487	0.00	0.8785	0.14
PC(40:5)/PC(40:4).1	0.9487	-0.39	0.7128	-0.15
PC(41:5)/PC(41:4)	0.9487	0.12	0.2345	-0.58
PE(34:6e)	0.9487	0.11	0.7209	0.13
PE(38:2)/PE(38:1)	0.9487	0.00	0.5054	-0.10
PE(38:4)/PE(38:3).1	0.9487	-0.24	0.2786	-0.47
PE(38:6)/PE(38:5).1	0.9487	0.03	0.1049	0.90
SM(d18:1/17:0)/SM(d18:1/15:0)	0.9487	-0.05	0.7984	-0.07
SM(d18:1/23:1)	0.9487	0.10	0.6742	0.37
TG(44:2)/TG(44:1)	0.9487	-0.06	0.0207	-0.37
TG(48:1)/TG(46:1)	0.9487	0.02	0.7209	0.19
TG(48:2)/TG(46:2)	0.9487	0.18	0.2786	-0.36
TG(52:0)/TG(50:0)	0.9487	-0.07	0.0830	-0.49
TG(52:1)/TG(50:1)	0.9487	-0.08	0.1605	0.31
TG(54:5)/TG(54:4)	0.9487	0.16	0.5054	0.32
TG(54:5)/TG(54:4).1	0.9487	0.16	0.2786	0.37
TG(58:9)/TG(56:9)	0.9487	0.16	0.2786	-0.22
PC(40:4e)	0.9738	0.20	0.0512	-0.39
TG(44:0)	0.9738	-0.05	0.0650	2.01
Cer(d18:1/11:0)	1.0000	0.22	0.9591	-0.09
LysoPE(20:0)	1.0000	-0.08	0.7924	-0.10
PA(36:4)	1.0000	0.00	0.3282	-0.06
PC(32:2)	1.0000	-0.16	0.4418	-0.04
PC(34:2)	1.0000	-0.02	0.7984	-0.16

PC(34:3)	1.0000	-0.09	0.4418	0.27
PC(38:3)/PC(36:3)	1.0000	0.05	0.1605	0.16
PE(36:0)/PE(34:0)	1.0000	-0.06	1.0000	0.02
PS(27:6)/PS(27:5)	1.0000	0.08	0.7209	0.01
PS(31:0e)	1.0000	0.10	0.4418	-0.13
TG(44:2)	1.0000	-0.19	0.1304	2.01
TG(46:2)	1.0000	-0.17	0.0650	1.84
TG(49:1)	1.0000	-0.05	0.0281	1.62
TG(52:3)	1.0000	-0.28	0.3823	0.25
TG(52:4)/TG(52:3)	1.0000	0.05	0.7209	0.02
TG(53:4)/TG(51:4)	1.0000	0.00	0.4418	0.09
